# Dual-Path Information Fusion and Twin Attention-Driven Global Modeling for Solar Irradiance Prediction

**DOI:** 10.3390/s23177469

**Published:** 2023-08-28

**Authors:** Yushi Yang, Zhanjun Tang, Zhiyuan Li, Jianfeng He, Xiaobing Shi, Yuting Zhu

**Affiliations:** 1 Faculty of Information Engineering and Automation, Kunming University of Science and Technology, Kunming 650500, China; yshiyang96@163.com (Y.Y.); lizy5733@gmail.com (Z.L.); jfenghe@kust.edu.cn (J.H.); sxb@stu.kust.edu.cn (X.S.); zyt@163.com (Y.Z.); 2 Key Laboratory of Artificial Intelligence in Yunnan Province, Kunming 650500, China

**Keywords:** solar irradiance forecasting, attention, deep learning, multi-step ahead

## Abstract

Accurate prediction of solar irradiance holds significant value for renewable energy usage and power grid management. However, traditional forecasting methods often overlook the time dependence of solar irradiance sequences and the varying importance of different influencing factors. To address this issue, this study proposes a dual-path information fusion and twin attention-driven solar irradiance forecasting model. The proposed framework comprises three components: a residual attention temporal convolution block (RACB), a dual-path information fusion module (DIFM), and a twin self-attention module (TSAM). These components collectively enhance the performance of multi-step solar irradiance forecasting. First, the RACB is designed to enable the network to adaptively learn important features while suppressing irrelevant ones. Second, the DIFM is implemented to reinforce the model’s robustness against input data variations and integrate multi-scale features. Lastly, the TSAM is introduced to extract long-term temporal dependencies from the sequence and facilitate multi-step prediction. In the solar irradiance forecasting experiments, the proposed model is compared with six benchmark models across four datasets. In the one-step predictions, the average performance metrics RMSE, MAE, and MAPE of the four datasets decreased within the ranges of 0.463–2.390 W/$m^{2}$, 0.439–2.005 W/$m^{2}$, and 1.3–9.2%, respectively. Additionally, the average $R^{2}$ value across the four datasets increased by 0.008 to 0.059. The experimental results indicate that the model proposed in this study exhibits enhanced accuracy and robustness in predictive performance, making it a reliable alternative for solar irradiance forecasting.

## 1. Introduction

As environmental pollution and the energy crisis worsen, focus on renewable energy is growing. Governments worldwide are prioritizing and supporting dependable and financially sustainable electric power systems. The International Energy Agency’s (IEA) 2023 Electricity Market Report reveals a nearly 11% growth in global renewable energy capacity in 2022, with solar capacity surging almost 18%. However, despite the growth in solar energy’s contribution to renewable energy, its penetration into the mainstream energy market remains modest [1]. This is primarily attributed to the inherent intermittency and instability caused by the fluctuating nature of solar radiation. Such volatility in solar photovoltaic (PV) systems significantly impacts the stability and reliability of the power supply, underscoring the need for enhanced predictability and security, while accurate forecasting of solar irradiance faces challenges in data acquisition and the influence of multiple variables plays a vital role in addressing these issues. Employing real-time electricity dispatch based on precise predictions of solar irradiance can enhance energy efficiency, stabilize the power supply, and reduce the costs of electricity [2].

In recent years, numerous models for predicting solar radiation have emerged, including physical models [3], statistical models [4], machine learning models, deep learning models [5], and hybrid models [6,7]. Physical models use solar radiation transfer equations and atmospheric principles to forecast solar radiation with meteorological data. For example, Angstrom [8] estimated total solar radiation at specific locations by analyzing the relationship between global solar radiation and sunshine duration. Meanwhile, Whillier [9] assumed constant atmospheric transmittance throughout the day and estimated hourly radiation values from daily radiation values through data analysis and theoretical derivation. However, these physical models encounter computational difficulties and have limited accuracy under diverse climatic conditions. In contrast, statistical models like ARIMA [10], SARIMA [11], VAR, and VARMA [12] use historical solar radiation data for forecasting. They handle time series data effectively, capturing trends and periodicity, as evidenced in previous work by Belmahdi et al. [13] and Shadab et al. [14]. The accuracy of these statistical models heavily depends on dataset quality and parameter selection, often overlooking the influence of external factors on solar radiation.

With the growing volume of data and advancements in machine learning, researchers have increasingly used neural network models to predict solar radiation. These methods mainly include both traditional machine learning and deep learning approaches. Among conventional machine learning techniques, commonly used methods include the support vector machine (SVM) [15,16], decision tree (DT) [17,18], artificial neural network (ANN) [19,20], and random forest (RF) [21,22]. However, these individual machine learning models often struggle to capture the complex patterns and nonlinear relationships in solar radiation time series data. This leads to suboptimal information utilization, incorrect feature selection, and significant prediction biases. To overcome these issues, researchers have combined optimization algorithms with machine learning models [23,24] for sequence forecasting. For example, Natgunanathan et al. [25] utilized RF algorithm and digital twin technology to predict and optimize power generation within the renewable energy microgrid project at the solar farm. Even though using optimization algorithms improves the feature selection capacity of individual machine learning models, they still face challenges with high-dimensional, large-scale, and complex-structured data. Furthermore, deep learning models have gained attention for their ability to automatically extract features, strong generalization capabilities, and expertise in handling large-scale data. For example, Kazem et al. [26] predicted the current and power of the grid-connected photovoltaic system (GCPV) using a full recurrent neural network (FRNN) combined with principal component analysis (PCA). In a similar vein, models like recurrent neural networks (RNN) [27,28], long short-term memory (LSTM) networks [29,30], transformer [31], and time convolutional networks (TCN) [32] are utilized in solar radiation time series research. Shekar et al. [33] conducted multi-step forecasting of solar radiation using the LSTM model. In a similar vein, Gao et al. [34] introduced the use of gated recurrent unit (GRU) for solar irradiance prediction. LSTM and GRU models, with their gating mechanisms and memory units, improve conventional RNNs’ ability to manage long sequences and dependencies. However, this complexity adds to computational demands, slowing down processing with intricate data such as solar radiation and possibly hindering the capture of continuous long-term trends.

Hybrid Models typically combine two or more different types of models [35,36]. The hybrid models primarily combine CNN, RNN, LSTM, and the attention mechanism, leveraging their unique advantages to enhance prediction accuracy. For example, Kumari et al. [37] demonstrated the effectiveness of an LSTM–CNN hybrid model for short-term global horizontal irradiance (GHI) prediction. Similarly, Zang et al. [38] introduced a cascaded CNN–LSTM structure for spatiotemporal correlation, using CNN for spatial feature extraction and LSTM for temporal dependencies. The cascaded structure improved understanding of spatial relationships and solar radiation time series data. To address the intermittency and instability of solar radiation, Gao et al. [39] utilized a combined CEEMDAN–CNN–LSTM model, achieving significant improvements in prediction performance, while the application of hybrid models is pivotal for solar radiation forecasting and shows promise, they also introduce complexity. Such complexity can result in slower processing speeds and challenges in capturing continuous long-term trends, especially with intricate data like solar irradiance.

Solar radiation’s dynamic interplay with meteorological variables results in unique traits. One notable characteristic is the overlapping of cycles caused by seasonal oscillations. This complexity imposes higher requirements on prediction models. Specifically, these models need to deal with intricate interrelations among multiple temporal intervals or spatial positions. Moreover, these models need to distribute attention across different features at various levels, thereby enhancing their capability to understand and identify complex relationships and patterns. The Attention Mechanism, which allows for the adaptive focus on different parts of a sequence, has been successfully integrated with deep learning models such as RNN and LSTM. This combination has shown the value of attention-based models in addressing these complexities, providing improved accuracy and efficiency. For example, Qin et al. [40] developed an approach that blends the time attention mechanism with RNN to capture the long-term temporal dependencies in time series data. Similarly, Pan et al. [41] integrated the attention mechanism with LSTM for the prediction of photovoltaic power generation. The integration of attention mechanisms augmented the prowess of the LSTM model for long-term sequence prediction. Aslam et al. [42] proposed a method that combines attention with LSTM, bolstering the model’s performance by assigning disparate weights to the importance of features. Zhang et al. [43] employed attention mechanisms with GRU for feature extraction, enabling a comparison of data before and after faults and capturing changes in different fault locations. The integration of attention mechanisms has helped to overcome some limitations of RNN and LSTM in feature modeling. However, the design of LSTM and GRU with gate structures introduces a new challenge. During sequence data processing, this model structure can lead to information loss. Consequently, critical information may be omitted, hindering the overall performance.

To address the aforementioned issues, this study propose a deep-learning-based solar radiation forecasting model, integrating three key components: a residual attention time convolution block (RACB), a dual-path information fusion module (DIFM), and a twin self-attention module (TSAM). Specifically, time series data typically contain multiple features, each with a potential impact on target value prediction. Although a correlation analysis is performed on the input data and some low-correlation features are removed, the existing feature selection method still falls short in accurately determining the significance of each feature. To tackle this issue, a feature attention is incorporated into RACB, enabling the network to adaptively discern the importance of each channel during the feature extraction process. By allocating different weights to different channels, the model effectively concentrates on the features most pertinent to the prediction target. Second, the DIFM comprises two parallel time series convolutional networks: the local feature extraction network (LFEN) and the dilated feature extraction network (DFEN). The LFEN is employed to extract local features from the time series, while the DFEN captures a wider context information by expanding the receptive field. By integrating features from both paths, the model is designed to boost its feature representation ability and strengthen its robustness against input data variations. Finally, seasonal fluctuations often lead to overlapping signals from different periods, complicating the model’s ability to extract effective feature representations. To effectively manage this situation, the model needs to learn long-term dependencies in the data. To address this issue, the TSAM is proposed, which encompasses channel self-attention and sequence self-attention. This module aims to globally model the features produced by the DIFM from both a sequence and a channel perspective, thereby enhancing the predictive capabilities of the model.

In summary, the contributions of this study encompass the following three aspects:

- A novel solar radiation forecasting model is proposed that innovatively incorporates a residual attention time convolution block (RACB). This design enables the model to selectively enhance significant features for irradiance prediction while diminishing the importance of irrelevant ones;
- A dual-path information fusion module (DIFM) is proposed, adeptly integrating both local features and broader contextual information. Through the consolidation of features from distinct scales, the module enhances the model’s representational capacity, thereby bolstering its robustness against variations in input data;
- A twin self-attention module (TSAM) is designed. By modeling long-distance dependencies in channels and sequences dimensions, the predictive capability of the model is improved. Experimental results on several public datasets demonstrate the effectiveness of the methods proposed in this study.

The remainder of this study is structured as follows: Section 2 describes the detailed structure of the model. In Section 3, the performance of the proposed model is evaluated through experiments on several publicly available datasets. Finally, in Section 4, a summary and discussion of the entire study is provided.

## 2. Methodology

### 2.1. Overview of the Proposed Method

**Figure 1:**
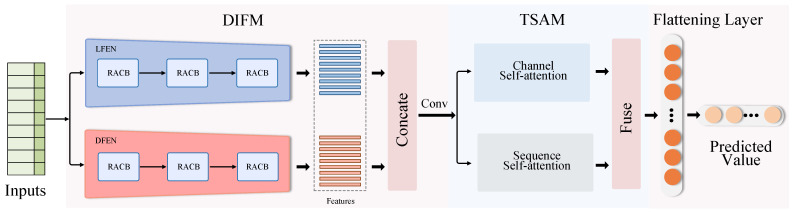
The overall structure of the proposed method.

To tackle the technical challenges in solar irradiance forecasting, this study proposes a method based on dual-path information fusion and twin self-attention for predicting solar irradiance. As depicted in Figure 1, the proposed method incorporates three key components: (1) the residual attention temporal convolution block (RACB), characterized by incorporating feature attention into the temporal convolution block, aimed at adaptively learning important features; (2) the dual-path information fusion module (DIFM) is designed to augment the model’s feature representation capacity by synergistically merging local features from distinct branches with wider contextual information; and (3) the twin self-attention module (TSAM), specifically formulated for global modeling, with an aim to forge long-distance dependencies across both channel and sequence dimensions, thereby contributing to enhanced prediction accuracy of the model.

### 2.2. Residual Attention Temporal Convolution Block

When carrying out time series prediction tasks, the importance of different features varies. These features impact the prediction results to different degrees, even though preliminary feature screening is conducted before inputting them into the network. However, the manually selected features still struggle to effectively balance the importance of each feature. To enhance the ability of convolutional networks to handle time series modeling and forecasting with a large number of features, this study designs a RACB. This module introduces a feature attention mechanism that can adaptively learn and understand the importance of each feature.

**Figure 2:**
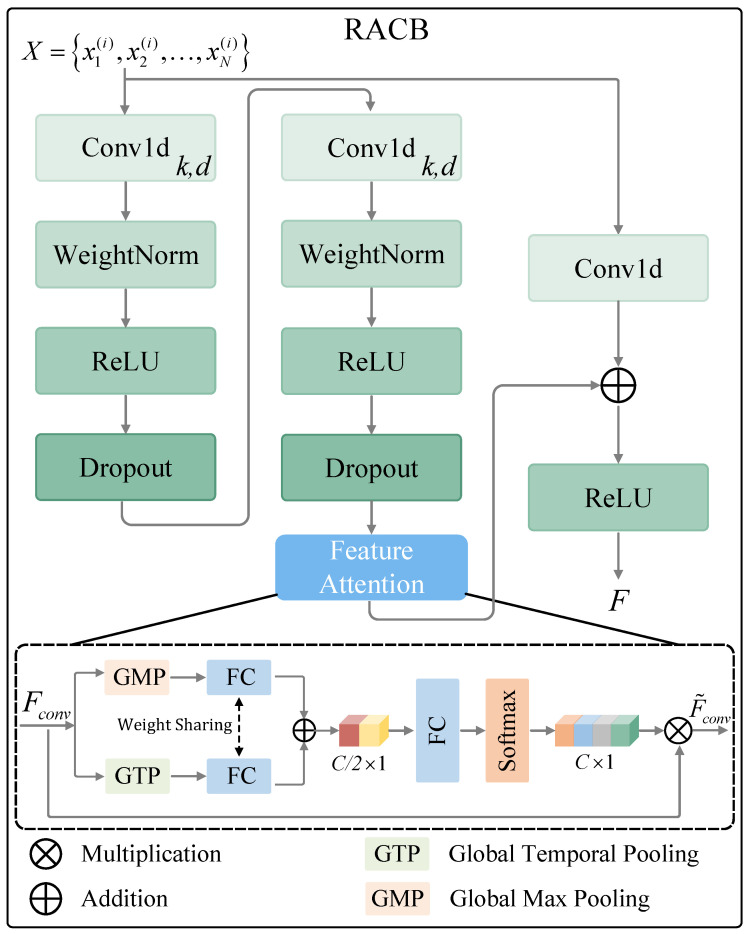
The proposed residual attention temporal convolution block (RACB), where *k* denotes the kernel size and *d* denotes the dilation factor.

Figure 2 presents the RACB, with the kernel size and dilation factor represented by *k* and *d*, respectively. Consider an input time series $X\in R^{C\times N}$, where *C* represents the number of features and *N* represents the sequence length. This model applies the RACB to extract features from *X*, which is defined as follows:

(1) $$F=\sigma \left(F_{attn}\left(F\left(X\right)\right)+F_{1\times 1}\left(X\right)\right)$$

where $X=\left\{x_{1}^{\left(i\right)},x_{2}^{\left(i\right)},\ldots ,x_{N}^{\left(i\right)}\right\}$, i∈{1,2,…,C}, F(·) signifies two 1D convolution layers, interspersed with WeightNorm, ReLU activation, and Dropout operations. σ denotes the ReLU activation function, $F_{1\times 1}\left(\;\cdot \;\right)$ refers to the 1 × 1 convolution operation, while $F_{attn}\left(\;\cdot \;\right)$ denotes the feature attention. Formally, let us assume that $F_{conv}\in R^{C\times N}$ represents the features output by the convolution layer F(·). The global max pooling function Fgmp(·) and the global temporal pooling function Fgtp(·) are employed to compress $F_{conv}$ into two low-rank tensors: $F_{gtp}\left(F_{conv}\right)\in R^{C\times 1}$ and $F_{gmp}\left(F_{conv}\right)\in R^{C\times 1}$, respectively. Subsequently, a fully connected layer $F_{FC}^{\alpha }$, which shares weights, is used to reduce dimensionality and refine useful information. By performing an addition operation, a tensor $\phi \in R^{C/2\times 1}$ is obtained, representing the global information of the original input $F_{conv}$. This can be formulated as follows:

(2) $$\phi =F_{FC}^{a}\left(F_{gmp}\left(F_{conv}\right)\right)+F_{FC}^{a}\left(F_{gtp}\left(F_{conv}\right)\right)$$

Next, a fully connected layer, denoted as $F_{FC}^{\beta }$, is employed to calculate the low-rank tensor $\eta \in R^{C\times 1}$, which is utilized to adjust the weights of the input features. This is defined as follows:

(3) $$\eta =F_{sfm}\left(F_{FC}^{\beta }\left(\phi \right)\right)$$

where $F_{sfm}\left(\;\cdot \;\right)$ represents the Softmax operation. The features, denoted as $F_{conv}$, are readjusted by η, as follows:

(4) $${\tilde{F}}_{conv}=\eta ⊙F_{conv}$$

where ⊙ denotes the multiplication and ${\tilde{F}}_{conv}\in R^{C\times N}$. In this way, the network is designed to adaptively focus on the features most informative for predicting solar irradiance, while suppressing those features that contribute less to the prediction.

### 2.3. Dual-Path Information Fusion Module

To capture both short-term and long-term patterns in time series, this study introduces a DIFM composed of a local feature extraction network (LFEN) and a dilated feature extraction network (DFEN). The LFEN is specifically designed to extract local features in the time series, while the DFEN is structured to capture a wider range of contextual information by increasing its receptive field. The features extracted by the LFEN are embedded into those extracted by the DFEN, achieving deep fusion of features at different scales. The structure of the LFEN, as depicted in Figure 1, comprises three cascaded RACBs. For a given input sequence $X\in R^{C\times N}$, the convolution kernel size k for each layer is fixed at 3, and the dilation factor d is fixed at 1. The entire process can be defined as follows:

(5) $$F_{LF}=F_{LFEN}\left(X\right)$$

where $F_{LFEN}\left(\;\cdot \;\right)$ denotes the LFEN. The structure of the DFEN is shown in Figure 1. It consists of three RACBs. In the RACB, dilated convolution is introduced to expand the receptive field of the convolution kernel, to better capture a wider range of contextual information in the sequence. The convolution kernel size k for each block is fixed at 2, and the dilation factors d are sequentially 1, 2, and 4. The entire process can be defined as follows:

(6) $$F_{DF}=F_{DFEN}\left(X\right)$$

where $F_{DFEN}\left(\;\cdot \;\right)$ denotes the DFEN. Subsequently, the features from both paths are fused as defined below:

(7) $$F_{fuse}=F_{1\times 1}\left(\left[F_{LF},F_{DF}\right]\right)$$

where [·,·] represents the concatenation operation, and $F_{1\times 1}\left(\;\cdot \;\right)$ denotes a 1×1 convolution operation.

### 2.4. Twin Self-Attention Module

**Figure 3:**
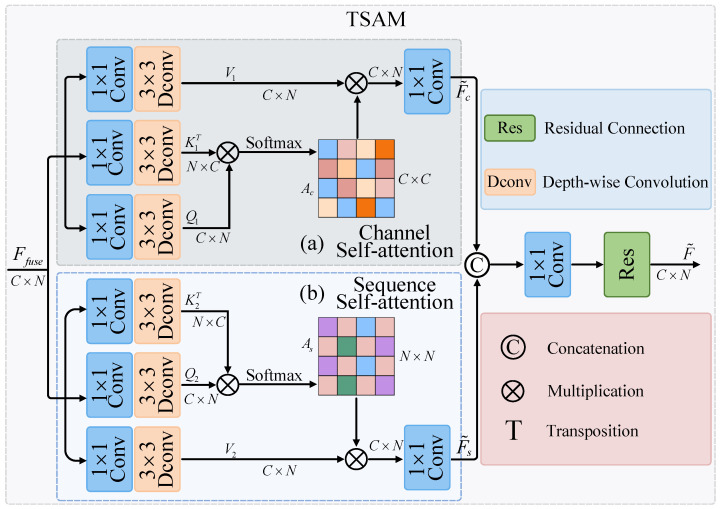
Architecture of the twin self-attention module (TSAM).

Through the DIFM, the multi-scale fusion feature $F_{fuse}$ is obtained. To further enhance the global context information, the TSAM is employed to model the feature $F_{fuse}$, which consists of two independent self-attention mechanisms: channel self-attention and sequence self-attention. Specifically, the channel self-attention is first introduced, as depicted in Figure 3a. For the input feature, to capture the global dependency on the channel dimension, 1×1 point-wise convolution and 3×3 depth-wise convolution are utilized to generate $Q_{1}=F_{DWC}^{Q_{1}}\left(F_{PWC}^{Q_{1}}\left(F_{fuse}\right)\right)$, $K_{1}=F_{DWC}^{K_{1}}\left(F_{PWC}^{K_{1}}\left(F_{fuse}\right)\right)$, and $V_{1}=F_{DWC}^{V_{1}}\left(F_{PWC}^{V_{1}}\left(F_{fuse}\right)\right)$, which encapsulate rich local context information. $F_{PWC}\left(\;\cdot \;\right)$ and $F_{DWC}\left(\;\cdot \;\right)$, respectively, represent point-wise convolution and depth-wise convolution. Then, a multiplication is performed on $Q_{1}$ and $K_{1}$ to generate the channel attention matrix $A_{c}\in R^{C\times C}$. The entire calculation process for channel self-attention is defined as follows:

(8) $${\tilde{F}}_{c}=F_{1\times 1}\left(\mathrm{Softmax}\left(Q_{1}\;\cdot \;K_{1}^{T}/\alpha_{1}\right)\;\cdot \;V_{1}\right)$$

where $F_{fuse}$ and ${\tilde{F}}_{c}$ are the input and output feature maps, respectively. $Q_{1}\in R^{C\times N}$, $K_{1}^{T}\in R^{N\times C}$, $V_{1}\in R^{C\times N}$. $\alpha_{1}$ is a learnable parameter used to control the magnitude of the output between $Q_{1}$ and $K_{1}$. To capture the global dependencies in the sequence dimension, sequence self-attention is introduced, as illustrated in Figure 3b. Another set of 1×1 point-wise convolution and 3×3 depth-wise convolution is used to generate $Q_{2}=F_{DWC}^{Q_{2}}\left(F_{PWC}^{Q_{2}}\left(F_{fuse}\right)\right)$, $K_{2}=F_{DWC}^{K_{2}}\left(F_{PWC}^{K_{2}}\left(F_{fuse}\right)\right)$, and $V_{2}=F_{DWC}^{V_{2}}\left(F_{PWC}^{V_{2}}\left(F_{fuse}\right)\right)$. Then, a multiplication is performed on $Q_{2}$ and $K_{2}$ to generate the sequence attention matrix $A_{s}\in R^{N\times N}$. The entire calculation process for sequence self-attention is defined as follows:

(9) $${\tilde{F}}_{s}=F_{1\times 1}\left(V_{2}\;\cdot \;\mathrm{Softmax}\left(K_{2}^{T}\;\cdot \;Q_{2}/\alpha_{2}\right)\right)$$

where $F_{fuse}$ and ${\tilde{F}}_{s}$ are the input and output feature maps, respectively. $Q_{2}\in R^{C\times N}$, $K_{2}^{T}\in R^{N\times C}$, $V_{2}\in R^{C\times N}$. $\alpha_{2}$ is a learnable parameter used to control the magnitude of the output between $Q_{2}$ and $K_{2}$. Subsequently, the features ${\tilde{F}}_{c}$ and ${\tilde{F}}_{s}$ are fused and combined with the initial input $F_{fuse}$. This operation can be formalized as follows:

(10) $$\tilde{F}=F_{1\times 1}\left(\left[{\tilde{F}}_{c},{\tilde{F}}_{s}\right]\right)+F_{fuse}$$

where [·,·] denotes the concatenation operation, and $F_{1\times 1}\left(\;\cdot \;\right)$ signifies a 1×1 convolution operation. By implementing the aforementioned processes, long-term dependencies across various positions within the sequence are effectively captured.

### 2.5. Loss Function

In this work, the mean squared error (MSE) is chosen as the loss function to optimize the model. The MSE loss function measures the average of the squared differences between the predicted values of the model and the true target values. It serves as a common metric to quantify prediction errors. The specific calculation formula is as follows:

(11) $$MSE=\frac{1}{n}\sum_{i=1}^{n}{\left(Y_{i}-{\hat{Y}}_{i}\right)}^{2}$$

where $Y_{i}$ represents the *i*-th element in the target sequence, ${\hat{Y}}_{i}$ denotes the corresponding element in the prediction sequence generated by the neural network, and *n* denotes the length of the sequence.

## 3. Experiment

### 3.1. Datasets and Data Processing

**Datasets:** The study utilizes a dataset from the National Solar Radiation Database (NSRDB), available at https://nsrdb.nrel.gov/ (accessed on 5 February 2023). To ensure comprehensive experimentation, data from four geographically distinct locations were selected, specifically from Busan, Karnataka, Nevada, and Yunnan. Each of these datasets includes solar irradiance data recorded at 30-min intervals. Table 1 provides detailed information about these datasets, including their geographical longitude and latitude, climatic conditions, dataset size, and data resolution.

**Figure 4:**
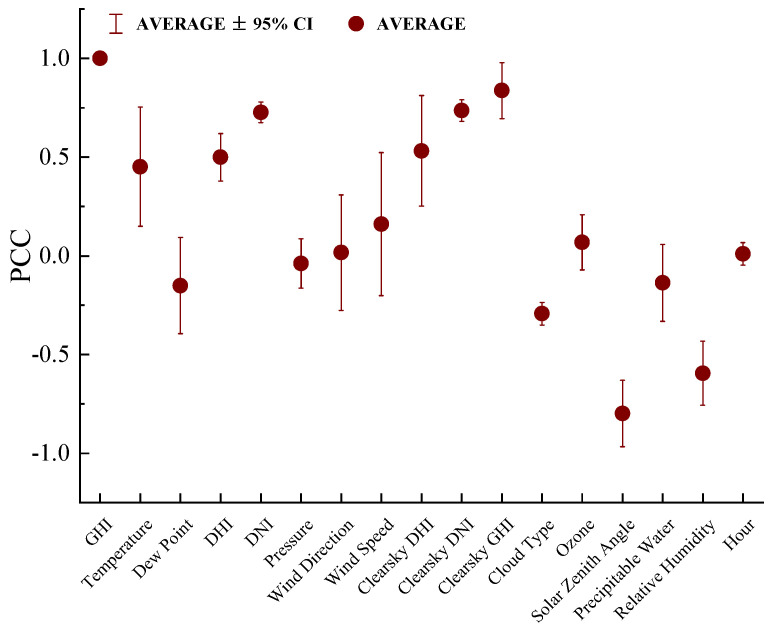
Pearson correlation value range plot between solar radiation and meteorological information.

**Data preprocessing:** A normalization process was applied to the solar irradiance time series to transform diverse value ranges into a uniform 0–1 range. The formula for this normalization is:

(12) $$f{\left(x_{i}\right)}_{\mathrm{norm}\;}=\frac{f\left(x_{i}\right)-f{\left(x_{i}\right)}_{\min}}{f{\left(x_{i}\right)}_{\max}-f{\left(x_{i}\right)}_{\min}}$$

where $f{\left(x_{i}\right)}_{\min}$ represents the minimum value in the data, $f{\left(x_{i}\right)}_{\max}$ denotes the maximum value, and $f\left(x_{i}\right)$ corresponds to the original value. Additionally, since the Global Horizontal Irradiance (GHI) is zero during the nighttime and does not require prediction, only data with GHI values greater than zero were retained. Moreover, to extract the features most closely related to solar irradiance from the dataset, the Pearson Correlation Coefficient (PCC) between solar irradiance and meteorological variables was calculated [44,45]. The value of the PCC lies between +1 and −1, where +1 signifies a perfect positive linear relationship between two variables, while −1 indicates a perfect negative linear relationship. Table 2 presents our results from the correlation coefficient calculation, reflecting the degree of correlation between each variable and GHI. The formula for calculating the PCC is as follows:

(13) $${\mathrm{PCC}}_{X,G}=\frac{E\left(\left(X-\mu_{X}\right)\left(G-\mu_{G}\right)\right)}{\sigma_{X}\sigma_{G}}$$

where ${\mathrm{PCC}}_{X,G}$ represents the Pearson correlation coefficient between variables *X* and *G*. The term *E* stands for expectation, whereas $\mu_{X}$ and $\mu_{G}$, respectively, denote the mean values of variables *X* and *G*. Additionally, $\sigma_{X}$ and $\sigma_{G}$ represent the standard deviations of variables *X* and *G*, respectively. The PCC was averaged across all four datasets [46], and features with an average value greater than 0.1 were considered as input features, as depicted in Figure 4. Ultimately, 13 features were selected as input: GHI, Temperature, Dew Point, DHI, DNI, Wind Speed, Clearsky DHI, Clearsky DNI, Clearsky GHI, Cloud Type, Solar Zenith Angle, Precipitable Water, and Relative Humidity.

### 3.2. Evaluation Metrics and Experimental Setup

**Evaluation metrics:** To provide a quantitative comparison of the proposed model, this study utilizes information from the first ’t’ moments to predict the GHI value at the ’t + n’ moment. Several metrics are employed, including the Root Mean Square Error (RMSE), Mean Absolute Error (MAE), Mean Absolute Percentage Error (MAPE), and R-squared ($R^{2}$), to facilitate an accurate analysis of the predictive outcomes.

The MAE represents the mean of the absolute differences between the predicted and actual values. The formula is:

(14) $$\mathrm{MAE}=\frac{1}{m}\sum_{i=1}^{m}\left|G_{i}-{\hat{G}}_{i}\right|$$

The RMSE is the square root of the mean of the squared differences between the predicted and actual values. A smaller RMSE value indicates a closer approximation of the predicted value to the actual one. The formula is:

(15) $$\mathrm{RMSE}=\sqrt{\frac{1}{m}\sum_{i=1}^{m}{\left(G_{i}-{\hat{G}}_{i}\right)}^{2}}$$

The MAPE is the mean of the absolute percentage errors between the predicted and actual values. A lower MAPE value indicates a closer approximation of the predicted value to the actual one. The formula is:

(16) $$\mathrm{MAPE}=\frac{100\%}{m}\sum_{i=1}^{m}\left|\frac{G_{i}-{\hat{G}}_{i}}{G_{i}}\right|$$

The $R^{2}$ quantifies the proportion of the variance in the dependent variable that can be predicted from the independent variable(s). A value closer to 1 signifies a better fit of the model to the observed data. The formula is:

(17) $$R^{2}=1-\frac{\sum_{i=1}^{m}{\left(G_{i}-{\hat{G}}_{i}\right)}^{2}}{\sum_{i=1}^{m}{\left(G_{i}-\bar{G_{i}}\right)}^{2}}$$

where $G_{i}$ represents the actual value of solar radiation irradiance, ${\hat{G}}_{i}$ denotes the predicted value of solar radiation irradiance, $\bar{G_{i}}$ is the mean of $G_{i}$, and *m* represents the total number of samples in the dataset.

**Figure 5:**
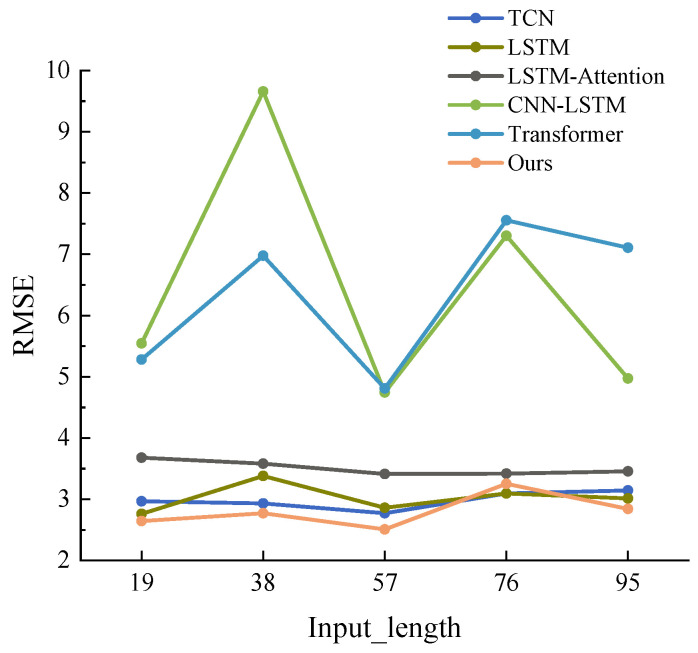
RMSE of various models for different input lengths.

**Experimental setup:** The algorithm in this study was developed using the PyTorch 1.12.1 framework and Python 3.8 on a server configured with a 12th Gen Intel (R) Core (TM) i9-12900K, an NVIDIA GeForce RTX 3090 GPU, and running the Ubuntu 20.04 LTS operating system. During the training process, the model parameters were optimized using the AdamW optimizer with a batch size of 256 and a learning rate of 0.001. The data was divided into training, validation, and test sets in a 6:2:2 ratio, and the model is trained for a total of 100 epochs. To identify the optimal length of the input sequence, various input sequence lengths were tested on the Nevada dataset to predict one-step GHI values. The RMSE values of each model served as the criterion for comparison. As shown in Figure 5, the figure depicts the RMSE values of one-step predictions of each model at different input lengths. Upon analysis of the results, it was determined that the prediction outcome was most optimal when the input length was 57. Consequently, the length of the input sequence was finalized as 57 for this study.

### 3.3. Result and Analysis

To evaluate the performance of the proposed model, systematic experiments were executed across distinct prediction intervals: 1, 2, 4, 6, 8, and 10 steps. Comprehensive insights can be derived from the experimental findings. The outcomes for the GHI time series prediction on the Busan dataset are delineated in Table 3. It is evident that the proposed approach consistently surpasses other models in predictive accuracy across varied intervals and evaluation criteria. For a one-step-ahead prediction, the model registered scores of 2.192 W/$m^{2}$, 1.517 W/$m^{2}$, 0.022, and 0.979 for RMSE, MAE, MAPE, and $R^{2}$, respectively. Noteworthy reductions in RMSE were observed when juxtaposed with models such as TCN [47], LSTM [48], LSTM-Attention [49], CNN-LSTM [50], and Transformer [51]. A discernible trend is that as the prediction horizon extends from 1 to 10 steps, there is a commensurate decline in the performance metrics for all models, underscoring the inherent challenge of forecasting over longer intervals. The Karnataka dataset’s results, tabulated in Table 4, further underscore the consistent superiority of the proposed model. In the context of a one-step prediction, the model’s performance metrics were impressive, and when compared against benchmarks like TCN, LSTM, and others, it exhibited noteworthy reductions in RMSE. For example, in the one-step-ahead prediction, the method yielded scores of 2.195 W/$m^{2}$, 1.503 W/$m^{2}$, 0.029, and 0.992 on the RMSE, MAE, MAPE, and $R^{2}$ metrics, respectively. Compared with TCN, LSTM, LSTM-Attention, CNN-LSTM, and Transformer, the model reduced the RMSE by 0.585 W/$m^{2}$, 0.518 W/$m^{2}$, 0.960 W/$m^{2}$, 3.283 W/$m^{2}$, and 5.880 W/$m^{2}$, respectively. The overarching observation from the table is the amplification of prediction errors as the forecasting range elongates, highlighting the intricacies associated with GHI forecasting. The results for the Nevada and Yunnan datasets are cataloged in Table 5 and Table 6. These findings further corroborate the proposed model’s exemplary predictive prowess over diverse horizons and metrics. Collectively, the empirical evidence affirms the model’s unparalleled capability in forecasting solar irradiance across varied temporal granularities, consistently outshining competitors across diverse datasets.

**Figure 6:**
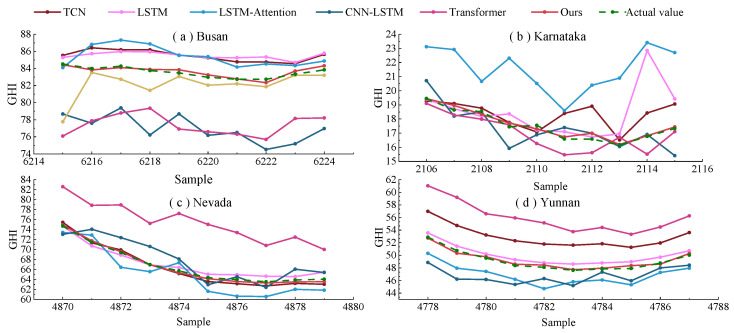
Prediction curve graphs of different prediction models one step ahead for four different datasets.

**Figure 7:**
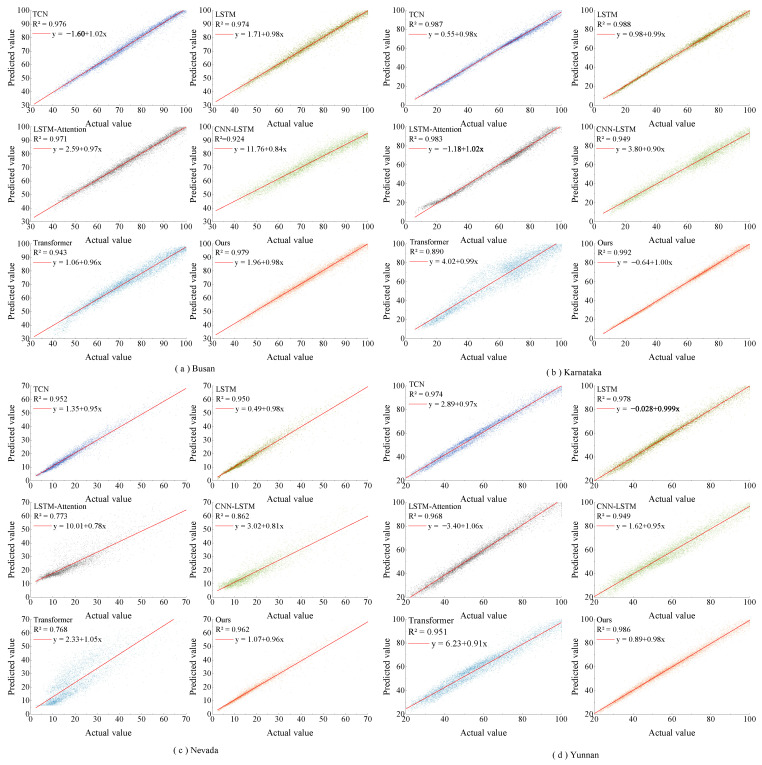
Scatter plots of predicted values and actual values one step ahead for different prediction models on four different datasets.

Figure 6 and Figure 7 show the one-step prediction results of various models across four datasets, represented as line graphs and scatter plots. Specifically, Figure 6 presents the prediction value curves for the four datasets. In contrast, Figure 7 displays the corresponding scatter plots of these prediction values. A detailed analysis of these charts yields several key observations: In the line graphs, the proposed model closely aligns with the actual irradiance curves, especially at inflection points where significant irradiance fluctuations occur. This highlights the strong fitting capability of the model. In the scatter plots, the predicted values from the model predominantly cluster around the diagonal line, signifying superior tracking performance. From the perspective of the datasets, the method effectively adapts to the unique characteristics inherent in each. The model maintains consistent predictive performance across different datasets, as evidenced in both line graphs and scatter plots. The prediction value curves showcase the efficiency of this model in forecasting solar irradiance fluctuations, and the scatter plots affirm its precision in capturing the linear trends within the data.

**Figure 8:**
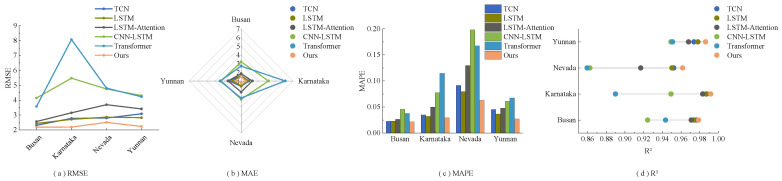
Performance evaluation metrics of various prediction models one step ahead for four different datasets.

**Figure 9:**
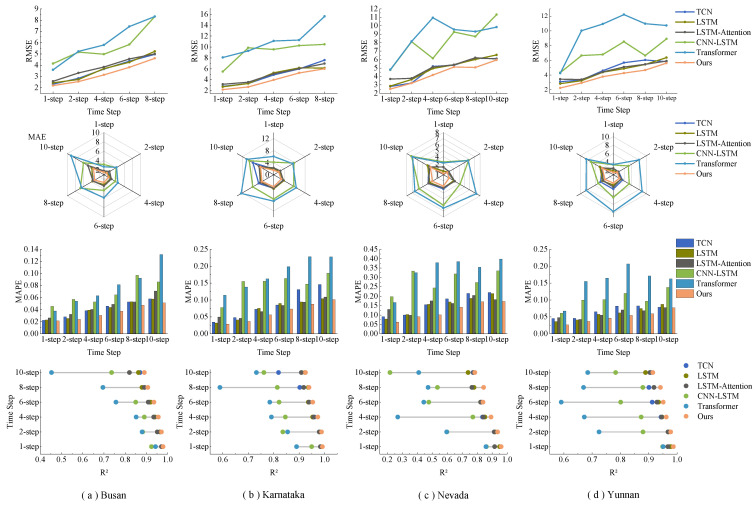
Performance evaluation metrics of different prediction models for multiple steps on four different datasets.

Figure 8 illustrates the predictive performance of various models across four different datasets. The RMSE line graph reveals that the proposed model consistently achieves the lowest RMSE values across all four datasets with minimal fluctuations. This indicates its superior error reduction performance. The MAPE bar chart shows that the proposed model records the smallest MAPE value for the Busan dataset and the largest for the Nevada dataset. Nonetheless, it consistently outperforms other models across all datasets. The $R^{2}$ graph demonstrates that the proposed model consistently records $R^{2}$ values near 1 across all datasets, surpassing other models in each instance. These findings collectively affirm the superior performance of the proposed model. Figure 9 illustrates the relationship between the model’s evaluation metrics and the change in prediction time step length. Despite the increase in MAE value as the prediction step length increases, the radar chart for MAE shows that the magnitude of change is relatively small, and the proposed model’s overall performance surpasses that of other models. Even in the Nevada dataset, where performance tends to be lower, the MAE value of the proposed model remains superior to those of other models. The MAPE bar chart clearly demonstrates that the proposed model maintains lower MAPE values across all four datasets, and achieves lower MSE and RMSE values in most instances. The lollipop chart for $R^{2}$ indicates that the proposed model consistently yields $R^{2}$ values closest to 1 across all datasets, suggesting a superior fitting capability compared to other models. In general, as the prediction time step length increases, the $R^{2}$ values of all six models decrease, and MSE, RMSE, and MAE values increase, indicating a decline in predictive performance. Nonetheless, the proposed model excels in most evaluation metrics, demonstrating consistently lower prediction errors, higher goodness-of-fit, and smaller percentage errors across various prediction time step lengths.

### 3.4. Further Exploration

**Figure 10:**
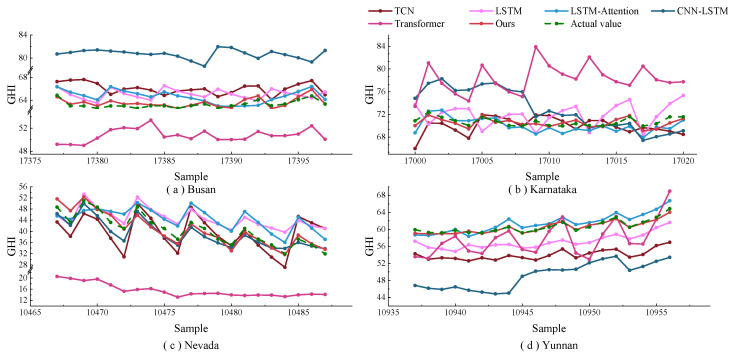
Prediction curve graphs of different prediction models four steps ahead for four different datasets.

**Figure 11:**
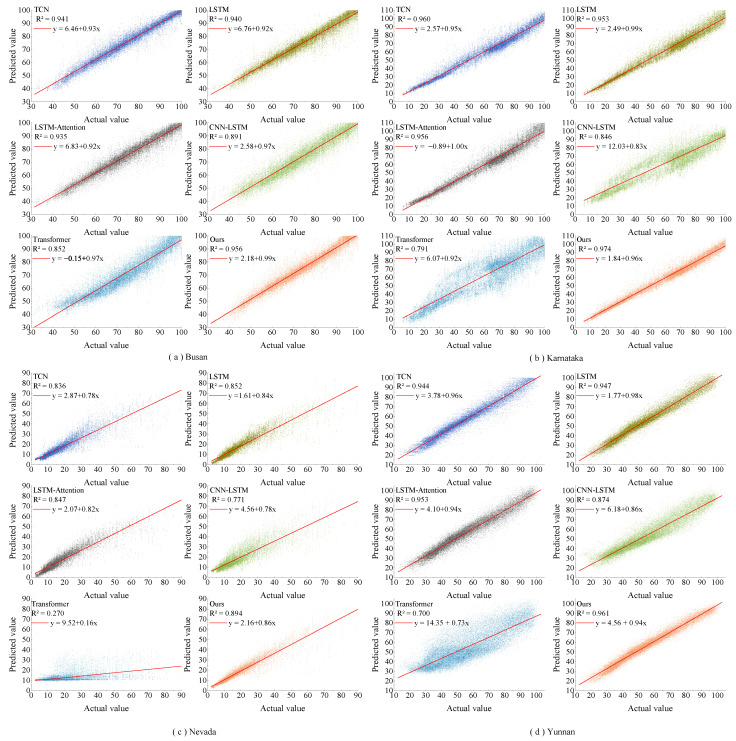
Scatter plots of predicted values and actual values four steps ahead for different prediction models on four different datasets.

To thoroughly evaluate the performance of the prediction model in a multi-step sequence prediction, analysis was conducted on a randomly selected fourth step. In the Busan dataset, the model delivered an RMSE of 3.144 W/$m^{2}$, an MAE of 2.144 W/$m^{2}$, a MAPE of 0.031, and an $R^{2}$ of 0.956. When pitted against algorithms like TCN, LSTM, LSTM-Attention, CNN-LSTM, and Transformer, the proposed model demonstrated a reduction in RMSE by 0.512 W/$m^{2}$, 0.545 W/$m^{2}$, 0.699 W/$m^{2}$, 1.832 W/$m^{2}$, and 2.651 W/$m^{2}$, respectively. For the Karnataka dataset, the proposed model achieved RMSE reductions of 0.922 W/$m^{2}$, 1.317 W/$m^{2}$, 1.131 W/$m^{2}$, 5.591 W/$m^{2}$, and 7.171 W/$m^{2}$. Similarly, in the Nevada and Yunnan datasets, the model exhibited RMSE reductions of 1.020 W/$m^{2}$, 0.764 W/$m^{2}$, 0.840 W/$m^{2}$, 1.962 W/$m^{2}$, 6.782 W/$m^{2}$, and 0.743 W/$m^{2}$, 0.623 W/$m^{2}$, 0.375 W/$m^{2}$, 3.033 W/$m^{2}$, and 6.698 W/$m^{2}$, respectively. Considering other performance indicators, the proposed model stood out, registering lower MAE and MAPE scores and attaining higher $R^{2}$ values. The prediction curves and scatter plots of this model across the four datasets are demonstrated in Figure 10 and Figure 11. Figure 10 displays the irradiance curves for these datasets, emphasizing particular time intervals. It is evident that the prediction model aligns remarkably with the actual curve, highlighting its superior forecasting capabilities for solar irradiance fluctuations. When benchmarked against other models, the proposed model shines in its tracking performance. It is particularly noteworthy during significant shifts, illustrating robust regression abilities and a negligible variance from actual values. This emphasizes its adeptness in utilizing time features. The scatter plots, as shown in Figure 11, for all datasets depict a close-knit distribution of the model’s predicted values around the diagonal. In comparison to other models, the proposed model demonstrates a denser distribution, closely trailed by the TCN and LSTM models. This further confirms the model’s exceptional proficiency in multi-step sequence predictions.

### 3.5. Ablation Study

In this subsection, an ablation study on the Busan dataset was undertaken to validate the efficacy of the introduced techniques. The findings, tabulated in Table 7, feature optimal results emphasized in bold. The table employs specific nomenclature for clarity. “w/o LFEN” indicates the removal of the LFEN, “w/o DFEN” represents the absence of the DFEN, “w/o RACB” signifies the omission of the RACB, “w/o TSAM” means the TSAM was excluded, and the term “Ours” is employed to denote the full-fledged model. All experiments were designed around a one-step prediction scenario. Upon evaluating the results, the RMSE values for the different models were discerned. The full model exhibited an RMSE of 2.19 W/$m^{2}$. In contrast, the “w/o LFEN”, “w/o DFEN”, “w/o RACB”, and “w/o TSAM” variants registered RMSEs of 2.540, 2.435, 2.313, and 2.467 W/$m^{2}$, respectively. Corresponding MAE values were documented as 1.517, 1.793, 1.680, 1.635, and 1.761 W/$m^{2}$. Concurrently, MAPE metrics stood at 0.022, 0.027, 0.025, 0.023, and 0.024, with $R^{2}$ values being 0.979, 0.972, 0.974, 0.976, and 0.973. A key observation from the one-step prediction outcomes is the RMSE’s sensitivity to the removal of any module. The variance ranged between 0.122 W/$m^{2}$ and 0.349 W/$m^{2}$. Notably, LFEN’s omission resulted in the most pronounced increase, highlighting its pivotal role in discerning local patterns within time-series datasets. Furthermore, in multi-step forecasts, the exclusion of TSAM consistently inflated the RMSE metric, underlining the significance of this module in enhancing the model’s predictive prowess.

**Figure 12:**
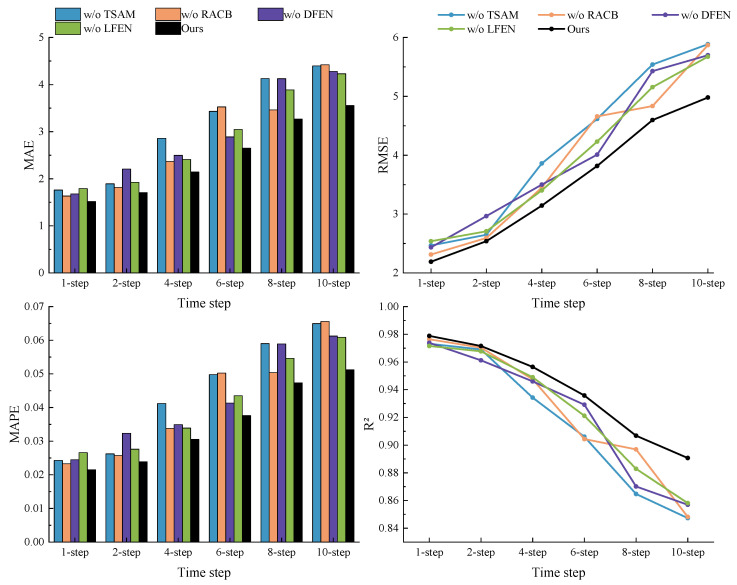
Performance evaluation metrics for ablation experiments.

Figure 12 depicts the fluctuation patterns of each model’s metric indicators as a function of prediction step length. The abscissa signifies the prediction step length, whereas the ordinate conveys the metric values. It is evident from the trends that, across different time scales, excluding any individual module leads to a surge in the RMSE, MAE, and MAPE values, while concurrently causing a decline in the $R^{2}$ value. Specifically, for one-step predictions, the omission of the LFEN escalates the MSE, MAE, and MAPE to their peak values, simultaneously dragging the $R^{2}$ to its lowest. Conversely, in the context of multi-step predictions, the absence of the TSAM results in maximal RMSE, MAE, and MAPE values, with the $R^{2}$ reaching its nadir.

## 4. Conclusions and Discussion

This study aims to develop a multi-step sequence prediction model for solar irradiance forecasting. To achieve this, a deep learning-based dual-path information fusion and twin attention-driven solar irradiance forecasting model is proposed. This model comprises three components: the RACB, the DIFM, and the TSAM. The RACB is designed to enable the network to adaptively learn important features while suppressing irrelevant ones. Following that, the DIFM is introduced to reinforce the model’s robustness against input data variations and integrate multi-scale features. Finally, the TSAM is introduced to extract long-term temporal dependencies within the sequence, thereby enhancing multi-step prediction capabilities.

The experimental results indicate that the model proposed in this study can accurately predict irradiance data. In both one-step and multi-step predictions, it significantly outperforms other models. Furthermore, when compared to other models such as TCN, LSTM, LSTM-Attention, CNN-LSTM, and Transformer, the proposed model exhibits superior and more robust performance across datasets from four different regions. For instance, for one-step predictions, the model reports RMSE values of 2.192 W/$m^{2}$, 2.195 W/$m^{2}$, 2.508 W/$m^{2}$, and 2.238 W/$m^{2}$ across the four datasets. When compared to algorithms such as TCN, LSTM, LSTM-Attention, CNN-LSTM, and Transformer, the proposed model achieves average RMSE reductions of 0.381 W/$m^{2}$, 0.212 W/$m^{2}$, 0.256 W/$m^{2}$, 1.052 W/$m^{2}$, and 1.855 W/$m^{2}$, underscoring its consistent performance across various datasets. For other multi-step predictions, the proposed model also surpasses other models, with the performance gap becoming more noticeable as prediction steps increase. Additionally, scatter plots and curve diagrams provide a more visual representation of the precision of the proposed method. Lastly, ablation studies were conducted to validate the effectiveness of the DFEN, the LFEN, the RACB, and TSAM. The results highlight that, when removing these components, there is a substantial decline in performance compared to the full model. For example, when LFEN is removed, RMSE increases from 2.19 W/$m^{2}$ to 2.540 W/$m^{2}$.

The prediction methods employed in this study have been summarized, and several conclusions are drawn. Firstly, solar irradiance is influenced by a multitude of factors, and the RACB effectively extracts essential features from this complex interplay. Secondly, these influencing factors exhibit distinct characteristics across different time scales. The DIFM captures these features across multiple scales, enhancing the model’s robustness and enabling precise predictions. Lastly, for extended-range forecasting, the TSAM addresses long-term dependencies in the data sequence, facilitating more accurate forecasting of future irradiance data. In contrast, other models like TCN, LSTM, and RNN do not consider the impacts of diverse factors and the importance of features across various scales. The model proposed in this study provides valuable support for photovoltaic power generation systems, a pivotal step toward the development of intelligent grid systems.

While the model excels in forecasting solar irradiance over multiple steps, there is room for refining its precision. Its primary reliance on past irradiance data might pose challenges in anticipating abrupt irradiance shifts. For even greater accuracy, future studies might consider broadening data sources and optimizing the neural network’s design. Addressing the intricate dynamics of irradiance patterns could demand meticulous feature engineering and model tweaks. Given the notable weather variations across different regions and seasons, it seems prudent to design models tailored to specific locales and times of the year. Such bespoke modeling, factoring in the unique climatic nuances of each area and season, stands to boost predictive accuracy.

## Figures and Tables

**Table 1:** General information of the four datasets.

Location	Latitude (${}^{\circ }$)	Longitude (${}^{\circ }$)	Size (Years)	Climate Type (Koppen Classification)	Data Resolution
Busan, South Korea	35.2101	129.0207	5	Temperate Monsoon	Half-Hourly
Karnataka, India	13.0745	77.5653	5	Tropical Monsoon	Half-Hourly
Nevada, United States	36.1811	−115.1346	3	Cold Desert	Half-Hourly
Yunnan, China	25.0918	102.9036	5	Temperate Monsoon	Half-Hourly

**Table 2:** Pearson correlation analysis of solar radiation and meteorological information.

Feature	Busan	Karnataka	Nevada	Yunnan	Average
GHI	1.000	1.000	1.000	1.000	1.000
Temperature	0.194	0.649	0.494	0.470	0.452
Dew Point	−0.026	−0.341	−0.028	−0.209	−0.151
DHI	0.582	0.525	0.403	0.487	0.499
DNI	0.760	0.725	0.739	0.683	0.727
Pressure	−0.019	0.049	−0.044	−0.141	−0.039
Wind Direction	0.064	−0.140	−0.114	0.256	0.016
Wind Speed	−0.083	0.068	0.202	0.453	0.160
Clearsky DHI	0.453	0.609	0.733	0.331	0.531
Clearsky DNI	0.726	0.767	0.760	0.693	0.736
Clearsky GHI	0.786	0.886	0.936	0.742	0.837
Cloud Type	−0.338	−0.263	−0.265	−0.305	−0.293
Ozone	0.182	−0.027	0.035	0.082	0.068
Solar Zenith Angle	−0.737	−0.847	−0.922	−0.688	−0.799
Precipitable Water	−0.152	−0.241	0.038	−0.192	−0.137
Relative Humidity	−0.553	−0.601	−0.491	−0.732	−0.594
Hour	−0.007	0.001	−0.017	0.062	0.010

**Table 3:** Forecasting performance of Busan Dataset.

Time Step	Evaluation Metrics	Model					
		TCN	LSTM	LSTM-Attention	CNN-LSTM	Transformer	Ours
1-step ahead	RMSE	2.314	2.441	2.569	4.147	3.583	**2.192**
	MAE	1.611	1.634	1.839	3.199	2.704	**1.517**
	MAPE	0.022	0.023	0.026	0.046	0.038	**0.022**
	$R^{2}$	0.976	0.974	0.971	0.924	0.943	**0.979**
2-step ahead	RMSE	2.836	2.730	3.314	5.152	5.219	**2.540**
	MAE	2.050	1.818	2.388	3.989	4.095	**1.705**
	MAPE	0.028	0.025	0.033	0.057	0.054	**0.024**
	$R^{2}$	0.965	0.967	0.952	0.883	0.880	**0.972**
4-step ahead	RMSE	3.656	3.689	3.843	4.976	5.795	**3.144**
	MAE	2.609	2.731	2.819	3.797	4.457	**2.144**
	MAPE	0.038	0.039	0.041	0.053	0.063	**0.031**
	$R^{2}$	0.941	0.940	0.935	0.891	0.852	**0.956**
6-step ahead	RMSE	4.355	4.258	4.555	5.835	7.425	**3.817**
	MAE	3.284	3.081	3.367	4.340	5.869	**2.649**
	MAPE	0.046	0.044	0.049	0.065	0.082	**0.038**
	$R^{2}$	0.916	0.920	0.909	0.850	0.757	**0.936**
8-step ahead	RMSE	4.966	5.226	4.993	8.317	8.307	**4.598**
	MAE	3.635	3.803	3.681	6.768	6.494	**3.266**
	MAPE	0.053	0.053	0.053	0.097	0.092	**0.047**
	$R^{2}$	0.891	0.880	0.890	0.695	0.696	**0.907**
10-step ahead	RMSE	5.420	5.587	6.384	7.733	11.138	**4.981**
	MAE	3.987	3.959	4.946	6.056	9.090	**3.554**
	MAPE	0.058	0.058	0.071	0.086	0.131	**0.051**
	$R^{2}$	0.871	0.863	0.820	0.737	0.453	**0.891**

**Table 4:** Forecasting performance of Karnataka Dataset.

Time Step	Evaluation Metrics	Model					
		TCN	LSTM	LSTM-Attention	CNN-LSTM	Transformer	Ours
1-step ahead	RMSE	2.780	2.713	3.155	5.478	8.075	**2.195**
	MAE	1.901	1.729	2.287	4.158	6.088	**1.503**
	MAPE	0.035	0.032	0.050	0.077	0.115	**0.029**
	$R^{2}$	0.987	0.988	0.983	0.949	0.890	**0.992**
2-step ahead	RMSE	3.289	3.308	3.557	9.839	9.271	**2.675**
	MAE	2.276	2.188	2.550	7.763	7.324	**1.856**
	MAPE	0.048	0.041	0.046	0.155	0.139	**0.037**
	$R^{2}$	0.982	0.982	0.979	0.836	0.855	**0.988**
4-step ahead	RMSE	4.870	5.265	5.079	9.539	11.119	**3.948**
	MAE	3.612	3.889	3.666	7.617	8.570	**2.894**
	MAPE	0.073	0.076	0.066	0.156	0.163	**0.056**
	$R^{2}$	0.960	0.953	0.956	0.846	0.791	**0.974**
6-step ahead	RMSE	5.986	6.160	6.028	10.266	11.277	**5.246**
	MAE	4.548	4.678	4.478	8.036	8.714	**3.943**
	MAPE	0.085	0.090	0.084	0.164	0.198	**0.074**
	$R^{2}$	0.939	0.936	0.939	0.822	0.785	**0.954**
8-step ahead	RMSE	7.590	6.143	6.961	10.478	15.596	**5.995**
	MAE	5.754	4.816	5.144	8.048	12.461	**4.522**
	MAPE	0.131	0.094	0.094	0.147	0.228	**0.088**
	$R^{2}$	0.903	0.936	0.918	0.814	0.589	**0.939**
10-step ahead	RMSE	10.332	6.751	7.353	11.863	12.589	**6.658**
	MAE	7.927	5.252	5.558	9.319	10.293	**5.000**
	MAPE	0.146	0.104	0.109	0.179	0.228	**0.101**
	$R^{2}$	0.820	0.923	0.909	0.762	0.732	**0.925**

**Table 5:** Forecasting performance of Nevada Dataset.

Time Step	Evaluation Metrics	Model					
		TCN	LSTM	LSTM-Attention	CNN-LSTM	Transformer	Ours
1-step ahead	RMSE	2.803	2.862	3.698	4.756	4.808	**2.508**
	MAE	1.602	1.569	2.277	3.115	2.965	**1.216**
	MAPE	0.091	0.080	0.130	0.198	0.167	**0.063**
	$R^{2}$	0.952	0.950	0.917	0.862	0.859	**0.962**
2-step ahead	RMSE	3.223	3.644	3.770	8.115	8.138	**3.205**
	MAE	1.813	2.007	2.031	5.796	5.693	**1.749**
	MAPE	0.101	0.103	0.100	0.334	0.325	**0.091**
	$R^{2}$	0.937	0.919	0.914	0.599	0.597	**0.938**
4-step ahead	RMSE	5.188	4.932	5.008	6.130	10.950	**4.168**
	MAE	3.034	2.966	3.080	4.166	7.292	**2.090**
	MAPE	0.155	0.158	0.170	0.245	0.379	**0.102**
	$R^{2}$	0.836	0.852	0.847	0.771	0.270	**0.894**
6-step ahead	RMSE	5.328	5.388	5.388	9.257	9.549	**5.122**
	MAE	3.354	3.197	3.151	6.042	6.509	**2.881**
	MAPE	0.186	0.170	0.163	0.318	0.385	**0.142**
	$R^{2}$	0.827	0.823	0.823	0.478	0.444	**0.840**
8-step ahead	RMSE	6.163	6.017	6.208	8.729	9.302	**5.064**
	MAE	3.864	3.588	3.854	5.626	6.463	**3.146**
	MAPE	0.216	0.189	0.204	0.273	0.355	**0.171**
	$R^{2}$	0.769	0.779	0.765	0.536	0.473	**0.844**
10-step ahead	RMSE	6.111	6.537	6.072	11.317	9.836	**5.935**
	MAE	3.840	4.052	3.615	7.106	6.879	**3.472**
	MAPE	0.221	0.214	0.182	0.335	0.398	**0.173**
	$R^{2}$	0.772	0.740	0.775	0.219	0.410	**0.785**

**Table 6:** Forecasting performance of Yunan Dataset.

Time Step	Evaluation Metrics	Model					
		TCN	LSTM	LSTM-Attention	CNN-LSTM	Transformer	Ours
1-step ahead	RMSE	3.086	2.825	3.416	4.309	4.232	**2.238**
	MAE	2.405	1.957	2.520	3.311	3.434	**1.526**
	MAPE	0.045	0.037	0.048	0.061	0.067	**0.027**
	$R^{2}$	0.974	0.978	0.968	0.949	0.951	**0.986**
2-step ahead	RMSE	3.329	3.223	3.382	6.634	10.025	**2.918**
	MAE	2.418	2.254	2.379	5.123	8.036	**2.074**
	MAPE	0.046	0.041	0.043	0.100	0.155	**0.037**
	$R^{2}$	0.970	0.972	0.969	0.879	0.725	**0.977**
4-step ahead	RMSE	4.505	4.385	4.137	6.795	10.460	**3.762**
	MAE	3.402	3.148	2.853	5.298	8.136	**2.623**
	MAPE	0.064	0.058	0.051	0.101	0.153	**0.047**
	$R^{2}$	0.944	0.947	0.953	0.874	0.700	**0.961**
6-step ahead	RMSE	5.678	4.858	5.077	8.532	12.219	**4.246**
	MAE	4.385	3.436	3.875	6.357	9.688	**2.981**
	MAPE	0.082	0.062	0.071	0.120	0.207	**0.055**
	$R^{2}$	0.912	0.935	0.929	0.801	0.591	**0.951**
8-step ahead	RMSE	6.044	5.429	5.439	6.643	10.976	**4.652**
	MAE	4.478	3.969	3.768	5.091	8.420	**3.270**
	MAPE	0.083	0.075	0.069	0.096	0.171	**0.060**
	$R^{2}$	0.900	0.919	0.919	0.879	0.670	**0.941**
10-step ahead	RMSE	5.821	6.367	5.897	8.905	10.733	**5.604**
	MAE	4.167	4.708	4.158	7.158	8.385	**4.041**
	MAPE	0.079	0.087	0.078	0.137	0.162	**0.077**
	$R^{2}$	0.907	0.889	0.905	0.783	0.685	**0.914**

**Table 7:** Evaluation metrics of ablation experiments.

Evaluation Metrics	Models	1-Step	2-Step	4-Step	6-Step	8-Step	10-Step
RMSE	w/o TSAM	2.467	2.649	3.861	4.617	5.540	5.886
	w/o RACB	2.313	2.594	3.450	4.658	4.836	5.870
	w/o DFEN	2.435	2.966	3.501	4.010	5.428	5.699
	w/o LFEN	2.540	2.708	3.403	4.231	5.156	5.674
	Ours	**2.191**	**2.540**	**3.144**	**3.817**	**4.598**	**4.981**
MAE	w/o TSAM	1.761	1.893	2.858	3.432	4.128	4.395
	w/o RACB	1.634	1.814	2.369	3.526	3.461	4.419
	w/o DFEN	1.680	2.207	2.497	2.888	4.125	4.279
	w/o LFEN	1.793	1.923	2.410	3.046	3.888	4.230
	Ours	**1.517**	**1.705**	**2.144**	**2.649**	**3.266**	**3.554**
MAPE	w/o TSAM	0.024	0.026	0.041	0.050	0.059	0.065
	w/o RACB	0.023	0.026	0.034	0.050	0.050	0.066
	w/o DFEN	0.025	0.032	0.035	0.041	0.059	0.061
	w/o LFEN	0.027	0.028	0.034	0.044	0.055	0.061
	Ours	**0.022**	**0.024**	**0.031**	**0.038**	**0.047**	**0.051**
$R^{2}$	w/o TSAM	0.973	0.969	0.934	0.906	0.865	0.847
	w/o RACB	0.976	0.970	0.948	0.904	0.897	0.848
	w/o DFEN	0.974	0.961	0.946	0.929	0.870	0.857
	w/o LFEN	0.972	0.968	0.949	0.921	0.883	0.858
	Ours	**0.979**	**0.972**	**0.956**	**0.936**	**0.907**	**0.891**

## Data Availability

The source code is available at https://github.com/89Mansions/DFTD, accessed on 21 August 2023.
